# An image cryptography method by highly error-prone DNA storage channel

**DOI:** 10.3389/fbioe.2023.1173763

**Published:** 2023-04-19

**Authors:** Xiangzhen Zan, Ling Chu, Ranze Xie, Yanqing Su, Xiangyu Yao, Peng Xu, Wenbin Liu

**Affiliations:** ^1^ Institute of Computational Science and Technology, Guangzhou University, Guangzhou, Guangdong, China; ^2^ School of Computer Science of Information Technology, Qiannan Normal University for Nationalities, Duyun, Guizhou, China; ^3^ Guangdong Provincial Key Laboratory of Artificial Intelligence in Medical Image Analysis and Application, Guangzhou, Guangdong, China

**Keywords:** image encryption, DNA storage, highly error-prone DNA storage channel, multiple sequence alignment, information security

## Abstract

**Introduction:** Rapid development in synthetic technologies has boosted DNA as a potential medium for large-scale data storage. Meanwhile, how to implement data security in the DNA storage system is still an unsolved problem.

**Methods:** In this article, we propose an image encryption method based on the modulation-based storage architecture. The key idea is to take advantage of the unpredictable modulation signals to encrypt images in highly error-prone DNA storage channels.

**Results and Discussion:** Numerical results have demonstrated that our image encryption method is feasible and effective with excellent security against various attacks (statistical, differential, noise, and data loss). When compared with other methods such as the hybridization reactions of DNA molecules, the proposed method is more reliable and feasible for large-scale applications.

## 1 Introduction

As the storage medium of genetic information, DNA molecules have the advantage of long durability, high density, and low cost. Recent advancements in their synthesis and sequencing technologies have made DNA a promising medium to deal with the challenges of data explosion (Qian et al., 2020; Meiser et al., 2022). Currently, researchers have devoted a lot of effort to accurately recover information from the noised sequence pool (Erlich and Zielinski, 2017; Meiser et al., 2019; Antkowiak et al., 2020; Press et al., 2020; Jeong et al., 2021). However, how to ensure the security of private data in DNA storage is an important question that is still in its infancy.

Clelland et al. (1999) first hid some secret letters in microdots of DNA molecules. Later, Gehani et al. (2004) realized the one-time pad encryption on DNA molecules through DNA microarray technology. In the past decade, researchers have continued to explore the encryption potential of complex biochemical processes. Yang et al. (2014) implemented a 32-bit one-time pad encryption that simulated one-bit exclusive-OR (XOR) operation by DNA strand displacement reaction (SDR). Later, Peng et al. (2018) developed a three-dimensional DNA self-assembly pyramid structure to achieve double-bit encryption. Zhang et al. (2019) constructed a DNA origami cryptography method by folding M13 viral scaffolds which could communicate braille-like patterns at the nanometer scale. Zakeri et al. (2016) accomplished short message communication by chromatogram patterning and multiplexed DNA sequence encoding technology. Peng et al. (2021) proposed a one-time-pad cipher algorithm by confusion mapping and random adapter, which could guarantee controllable biological security. Recently, some researchers also developed an SDR-based chaos system to generate secret keys (Wang et al., 2020; Zou et al., 2021; Zhu et al., 2022). However, the reliability and practicability of these methods are limited in two aspects. First, they are vulnerable to the base errors that are prevalent in DNA storage. Due to the over-reliance on highly specific biomolecule hybridization reactions, these methods require specialized design and accurate synthesis of DNA sequences. Even a few base errors can cause encryption failure. Second, the experiments are sophisticated and may produce unpredictable results in case of some subtle variations in experiment conditions (temperature, time, and ion concentration). Moreover, noise environments may even worsen the unpredictability of the results. In addition, these experimental processes are time-consuming, difficult to monitor, and not suitable for large-scale applications.

**FIGURE 1:**
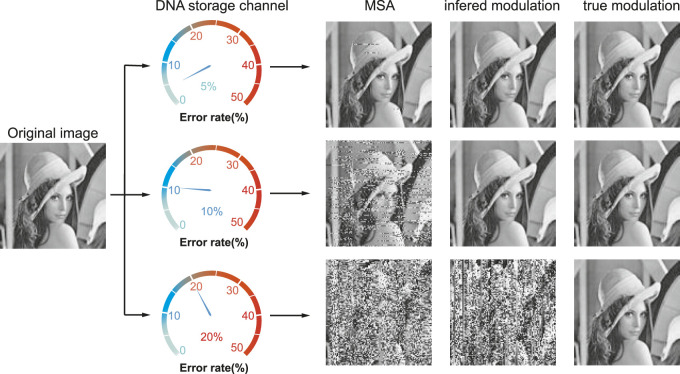
Recovered images at error rate 5%, 10%, and 20% by MSA, inferred, and true modulation signal. The recovered images by the first two methods gradually become vague as the error rate increases, while the one by the true modulation signal is completely correct.

Recently, our group proposed a modulation-based DNA storage architecture that is extremely robust to insertion–deletion–substitution (IDS) errors. The basic idea is that the modulation signal not only converts the binary information into DNA sequences during the write phase but also detects synchronization errors and decodes the corrected data during the read phase (Zan et al., 2022). Figure 1 shows an example of the recovered image under different noise levels by three strategies. The first one reconstructs the images directly by multiple sequence alignment (MSA) algorithms. The second one infers a possible modulation signal *M*′ by MSA and then reconstructs the images using the inferred *M*′ as in Zan et al. (2022). While the last one recovers images using the true modulation signal *M*. As noises increase, the first two gradually fail to recover the original image while the last one could perfectly recover it. Since MSA is the only method available for noise correction without the knowledge of coding in DNA storage and since IDS errors are inherent in the synthesis and sequencing processes, the modulation signal could serve as a secure key in a high-error DNA storage channel.

In this article, we explore the feasibility of image encryption in a high DNA storage channel. The proposed image encryption scheme consists of two layers: conventional encryption and DNA storage channel encryption. The first layer implements pixel scrambling and diffusion, and the second layer adds further complex confusion to DNA sequences (or DNA pixels) by taking advantage of the uncertainty in the DNA storage channel. Simulation results have demonstrated that the proposed method could resist cipher attack at the DNA sequence level when the noise is larger than 20%. It is also very robust to DNA base errors and sequence losses. Security analysis proves that it has a large key space, is sensitive to the key and plaintext, and can cope with statistical attacks. In sum, the proposed method achieves an excellent combination of the silico-based and carbon-based information security technologies and paves a solid foundation for data security in future DNA-based information architecture.

## 2 Encryption and decryption

**FIGURE 2:**
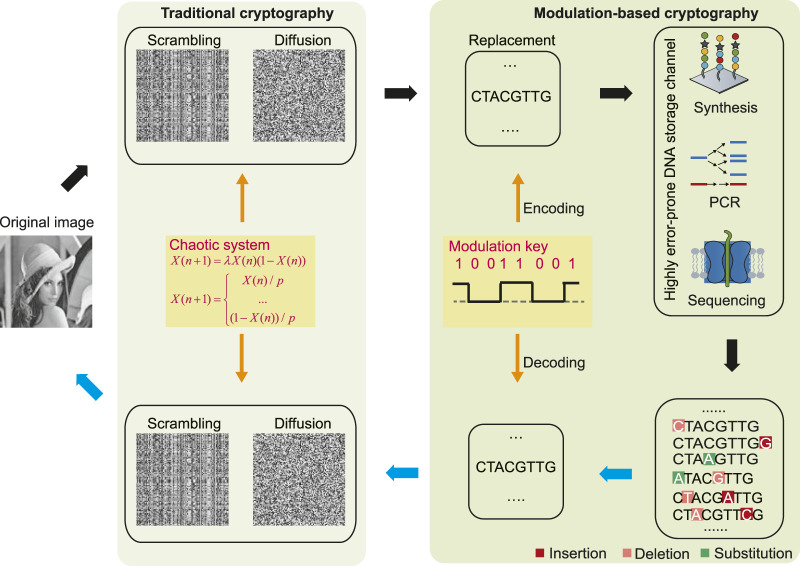
Schematic diagram of encryption and decryption processes. To the left is traditional cryptography which includes scrambling and diffusion at the binary level. To the right is modulation-based cryptography at the highly error-prone DNA storage channel. Black arrows represent the encryption process, while blue arrows indicate the decryption process.

Figure 2 shows the schematic diagram of the proposed encryption and decryption processes, which includes two stages. The first stage performs regular pixel scrambling and diffusion at the binary level. The second stage further encrypts the binary data into DNA sequences by a known modulation key; then, these are transmitted through the highly error-prone DNA storage channel, which consists of several DNA operating technologies with high error rates, such as light-directed maskless array DNA synthesis with an error rate of approximately 15% (Antkowiak et al., 2020), biased polymerase chain reaction (PCR) (Chen et al., 2020), and nanopore sequencing with error rates between 10% and 15% (Wang et al., 2021). Finally, the output ciphertext is a pool of DNA sequences involving large amounts of insertion–deletion–substitution errors. The decryption process is the reverse of encryption.

### 2.1 Secret key generation

Secret keys mainly consist of two parts. One is the chaotic systems which include the piecewise linear chaotic map (PWLCM) (Alawida et al., 2019; Zhou et al., 2021) and logistic map (Sui et al., 2015; 2014), while the other is the modulation key.

The dynamic equation of PWLCM can be described by the following function:

$$X\left(n+1\right)=\left\{\begin{array}{c} X\left(n\right)/p,0\leq X\left(n\right)<p\\ \left(X\left(n\right)-p\right)/\left(0.5-p\right),p\leq X\left(n\right)<0.5\\ \left(1-X\left(n\right)-p\right)/\left(0.5-p\right),0.5\leq X\left(n\right)<1-p\\ \left(1-X\left(n\right)\right)/p,1-p\leq X\left(n\right)\leq 1 \end{array}\right.$$ (1)

where the parameter *p* should be in the range of (0, 0.5), and the status value *X*(*n*) is in the range of (0,1).

The logistic map is defined as follows:

$$X\left(n+1\right)=\lambda X\left(n\right)\left(1-X\left(n\right)\right)$$ (2)

where the parameter *λ* should be in the range of (0, 4), and the status value *X*(*n*) is in the range of (0,1).

We use the abovementioned chaotic systems to generate three random sequences, two of which are generated by the PWLCM with the initial status values *X*_*r*_(0) and *X*_*c*_(0) and one by a logistic map with the initial status value *X*_*d*_(0). To relate the initial values with the plain image, we use Keccak (Bertoni et al., 2013) to hash the plain image to generate a fixed-length *K* (512 bit), which can be divided into 32 blocks, each of 16-bit. We denote it as *K* = {*k*_1_, *k*_2_, … , *k*_32_}. The initial status values are derived as follows:

$$\left\{\begin{array}{c} X_{r}\left(0\right)=\frac{1}{16}\overset{k_{11}}{\underset{i=k_{1}}{\sum }}\left(\frac{32}{4k_{i}+1}+\frac{1}{4k_{i}+3}+\frac{128}{10*k_{i}+5}+\frac{64}{10k_{i}+7}+\frac{4}{10k_{i}+9}+\frac{1}{10k_{i}+3}\right)\\ X_{c}\left(0\right)=\frac{1}{16}\overset{k_{22}}{\underset{i=k_{12}}{\sum }}\left(\frac{32}{4k_{i}+1}+\frac{1}{4k_{i}+3}+\frac{128}{10*k_{i}+5}+\frac{64}{10k_{i}+7}+\frac{4}{10k_{i}+9}+\frac{1}{10k_{i}+3}\right)\\ X_{d}\left(0\right)=\frac{1}{16}\overset{k_{32}}{\underset{i=k_{23}}{\sum }}\left(\frac{32}{4k_{i}+1}+\frac{1}{4k_{i}+3}+\frac{128}{10*k_{i}+5}+\frac{64}{10k_{i}+7}+\frac{4}{10k_{i}+9}+\frac{1}{10k_{i}+3}\right) \end{array}\right.$$ (3)

After retrieving the initial value [i.e., *X*_*r*_ (0)] and the corresponding chaotic map, we iterate through the chaotic map *n* times [(i.e., *X*_*r*_(*n*)] to remove transient processes and then continue to iterate it to obtain the random sequence of the specified length.

Modulation key *M* is a binary sequence of equal length to the encoded DNA sequence. In *M*, ‘0’ represents A/T and ‘1’ represents C/G (Zan et al., 2022). The 01 composition of the key directly reflects the base composition of the encoded DNA sequence. Since the DNA sequences with extreme guanine–cytosine (GC) content or long homopolymers (i.e., longer repeats of the same base, i.e., AAAAAA…) are difficult to synthesize and prone to sequencing errors, most of the DNA storage works comply with some encoding constraints on the DNA sequences, such as the GC content of 45%–55% and homopolymer runs of ≤3 nt (Erlich and Zielinski, 2017). In our encryption scheme, the percentage of 1 s (or 0 s) in the modulation key (equivalent to the GC content) and the consecutive length of 1 s (or 0 s) (equivalent to the homopolymer runs) also adhere to these constraints. From a key space perspective, this makes key cracking more difficult.

### 2.2 Encryption algorithm

Given an image *P* with size *W* × *H* and the iteration number $n\in \left[100,\infty \right)$ . Let *N* = *W* × *H*, the detailed encryption process can be depicted as follows.

#### 2.2.1 Traditional cryptography by scrambling and diffusion

Step 1 Get the secret keys *λ*, *p*, *X*_*r*_(0), *X*_*c*_(0), and *X*_*d*_(0).

Step 2 Use *X*_*r*_(0), *n*, and Eq. 1 to generate one sequence *S*_*R*_ of length *W*. Sort *S*_*R*_ in the ascending order to get the corresponding index sequence $S_{R}^{\prime }$ , number the rows of pixels of the original image *P*, and adjust row positions according to $S_{R}^{\prime }$ to finish row-wise permutation operations. The row-wise scrambled image is denoted as *P*_1_. For example, let *S*_*R*_ = {35, 60, 13} and image *P* = {*r*_1_, *r*_2_, *r*_3_}, where *r*_*i*_ (1 ≤ *i* ≤ 3) stands for the *i*-*th* row of pixels, the corresponding index sequence is $S_{R}^{\prime }=\left\{3,1,2\right\}$ , and the row-wise scrambled image is *P*_1_ = {*r*_3_, *r*_2_, *r*_1_}.

Step 3 Similar to Step 2, use *X*_*c*_(0), *n*, and Eq. 1 to generate one sequence *S*_*C*_ of length *H* and perform column-wise permutation operations on *P*_1_. The scrambled image is denoted as *P*_2_.

Step 4 Use *X*_*d*_(0), *n*, and Eq. 2 to generate a sequence *D* of length of *W* × *H*. Reshape *P*_2_ into one-dimensional sequence *Q*. Performing diffusion operation on *Q* using Eq. 4 yields *Q*′. Finally, reshape *Q*′ into a two-dimensional *W* × *H* matrix *P*_3_.

$$Q^{\prime }\left(i\right)=\left\{\begin{array}{c} \left(Q\left(1\right)⊕Q\left(N\right)⊕Q\left(N-1\right)⊕D\left(1\right)\right)mod256,\;i=1\\ \left(Q\left(2\right)⊕Q^{\prime }\left(1\right)⊕Q\left(1\right)⊕D\left(1\right)\right)mod256,\;i=2\\ \left(Q\left(i\right)⊕Q^{\prime }\left(i-1\right)⊕Q^{\prime }\left(i-2\right)⊕D\left(i\right)\right)mod256,i\in \left[3,N\right] \end{array}\right.$$ (4)

#### 2.2.2 Dynamic modulation cryptography

Step 1 Obtain the secret key *M*.

Step 2 Transform *P*_3_ into the binary form $P_{3}^{\prime }$ , and partition $P_{3}^{\prime }$ into strands of fixed length (*l* = *len*(*M*)). All these strands are encrypted with *M* to generate their corresponding DNA sequences *C* according to a simple mapping rule (00 → A, 01 → T, 10 → C, 11 → G). For instance, assuming *M* = ‘**1001**1001**1001**’, the message strand ‘010011010110’ is aligned with *M* into two rows, and a DNA sequence ‘**CTAC**GTAG**CTTC**’ can be obtained after mapping each column of the two rows into one DNA base.

Step 3 Transform *C* into the final ciphertext *C*′ through the highly error-prone DNA storage channel.

### 2.3 Decryption algorithm

As an asymmetric cryptosystem is more secure than a symmetric cryptosystem (Dong, 2015), the decryption keys are not identical to the encryption ones in our method. The decryption scheme uses the keys *λ*, *p*, *X*_*r*_(*n*), *X*_*c*_(*n*), *X*_*d*_(*n*), and *M* to execute the reverse operation on the encryption algorithm. First, according to the modulation decoding method (Zan et al., 2022), *M* is used to correct noises in the sequenced data *C*′ and decode them to obtain the two-dimensional pixel matrix *P*_3_. Second, Eqs. 4, 2, *λ*, and *X*_*d*_(*n*) are used to perform reverse diffusion operations on *P*_3_ to get *P*_2_. Finally, Eq. 1, *p*, *X*_*r*_(*n*), and *X*_*c*_(*n*) are used to perform reverse scrambling operations on *P*_2_ to derive plain image *P*.

## 3 Results

We demonstrate our results on the 100 × 100 Lena image as a proof of concept. It is encoded by 400 DNA sequences of 200 bases without considering overheads of the index because we assume that the clustering accuracy can be perfect. To investigate the proper noise channel for robust encryption, we take a series of simulation experiments with noises ranging from 2% to 40% and sequence copies ranging from 5 to 10,000.

### 3.1 Key space analysis

The key space of the proposed method is sufficiently large to withstand any brute force attack. In the traditional decryption process, the receiver has to know the five parameters *λ*, *p*, *X*_*r*_(*n*), *X*_*c*_(*n*), and *X*_*d*_(*n*). As their valid precision is 10^–16^, the key space of the five parameters will be

$$S_{\mathrm{key}}=10^{80}\approx 2^{266}$$ (5)

Given that the sequence length is 200, and the percentage of 1s in the carrier strand is about 0.5, the modulation key space is

$$S_{M}=\left(\begin{array}{c} 200\\ 100 \end{array}\right)\approx 2^{196}$$ (6)

The total key space of our method is

$$S=S_{\mathrm{key}}\times S_{M}=10^{80}\times \left(\begin{array}{c} 200\\ 100 \end{array}\right)\approx 2^{462}\gg 2^{128}$$ (7)

It is much larger than the theoretical secure key value 2^128^ (Dong et al., 2022). As the modulation key space alone is larger than 2^128^, we can conclude that the storage channel can serve as another layer for data security.

### 3.2 Ciphertext attack in DNA sequence level

Attackers have two possible ways to decipher the encrypted image in the noisy DNA storage channel. One is to infer a possible modulation key *M*′ by MSA and then decipher the sequenced reads by it, and the other is to directly decipher sequenced reads by the MSA algorithm. This is because there are only two methods of correcting base errors in DNA storage: constraint coding and multiple sequence alignment (MSA) without prior knowledge. As MSA fundamentally relies on pairwise sequence alignment algorithms, such as the Needleman–Wunsch algorithm (Needleman, 1970) and seeks to find a globally optimal alignment between multiple copies, there is limited variability in alignment accuracy across MSA software tools (Pervez et al., 2014). Assuming all keys are known except for *M*, we apply one of the famous MSA tools named MAFFT (Katoh et al., 2002) to conduct a series of experiments.

**FIGURE 3:**
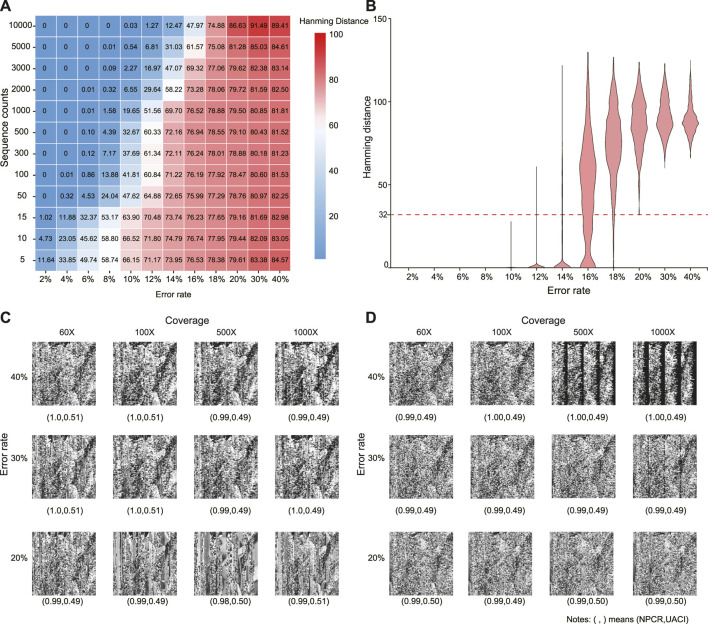
Cipher attack at different error rates and sequence copies. **(A)** Average Hamming distance between inferred and true modulation keys. **(B)** Distribution of Hamming distances of inferred modulation keys at sequence copies 10,000. **(C)** Decrypted images by inferred modulation keys. **(D)** Decrypted images by MSA. In **(C)** and **(D)**, the values in parentheses denote NPCR and UACI, respectively.

It is impossible to infer a potential modulation key when the error rate is higher than 20%. The attacker can decipher the encrypted image if the inferred key *M*′ is very similar to *M*. Here, we assume that the attackers could have sufficient sequence copies to infer *M*. Figure 3A shows that the average Hamming distance between *M* and *M*′ increases as the error rate increases. When the error rate is larger than 20%, the average Hamming distance is about 80, and increasing sequence copies may even result in a larger Hamming distance (see the top left corner). Figure 3B further shows the Hamming distance distribution at 10,000 sequence copies. The least Hamming distance may reach 32 at an error rate of 20%. That is, there are at least 32 errors in the inferred modulation keys with 200 bits. As the error rate increases, this lower limit could further increase. Therefore, inferring the true modulation key becomes almost impossible in a high error channel.

Without knowing the modulation key *M*, it is almost impossible to decipher the real image when the error rate is larger than 20%. To evaluate the difference between the decrypted and original images, the number of pixels change rate (NPCR) and unified average changing intensity (UACI) are calculated as

$$D\left(i,j\right)=\left\{\begin{array}{c} 1,c_{1}\left(i,j\right)\neq c_{2}\left(i,j\right)\\ 0,otherwise \end{array}\right.$$ (8)

$$NPCR=\frac{\sum_{ij}D\left(i,j\right)}{W\times H}\times 100$$ (9)

$$UACI=\frac{1}{W\times H}\left[\underset{ij}{\sum }\frac{\left|c_{1}\left(i,j\right)-c_{2}\left(i,j\right)\right|}{255}\times 100\%\right]$$ (10)

where *W* and *H* are the width and height of two images (*c*_1_ and *c*_2_), respectively. Figures 3C, D show the decrypted images using different sequence copies by the inferred modulation key and MSA, respectively. Compared with the original image, the decrypted images are all seriously distorted with *NPCR* ≈ 1 and *UACI* ≈ 0.5, even at sequence copies 1,000. The utilization of the traditional cryptographic techniques further increases crack difficulties.

### 3.3 Sensitivity analysis

The proposed method is sensitive to secrete keys and plaintext. A slight change in the key (i.e., a single bit change) or plaintext could cause a completely different encrypted result. First, the sensitivity of the PWLCM and logistic map has been confirmed in many image-encryption works (Sui et al., 2015; Sui et al., 2014; Alawida et al., 2019; Zhou et al., 2021). At the same time, 1 bit insertion/deletion in the modulation signal will affect the encoding of a large number of pixels. Second, plaintext sensitivity is accomplished by the pixel diffusion process and initial status values of the chaotic systems which are strongly related to the plain image.

### 3.4 Statistical analysis

**FIGURE 4:**
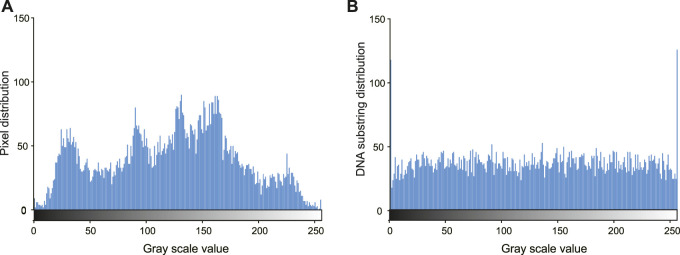
Distribution of pixel intensity histogram. **(A)** Plain image. **(B)** Encrypted image.

The proposed method can resist statistical attacks. Figure 4 shows the histogram of the pixels in the original image (A) and the encoded eight-base pixel DNA strands (B). The distribution of the encoded DNA sequences is more flat than that of the original. Considering the IDS noises in the sequenced reads, the distribution in (B) tends to be more uniform. Table 1 shows the correlation coefficients of the ciphered image after dislocation and diffusion. All values in the three directions are close to the ideal value of 0 (Ghadirli et al., 2019). That is, the encrypted pixels are distributed randomly. The information entropy of the cyphered image is 7.950121813, which is very close to the ideal value of 8 (Ghadirli et al., 2019). Therefore, the encrypted image shows favorable randomness.

**TABLE 1:** Correlation coefficients in different directions of original and ciphered images.

Image	Horizontal	Vertical	Diagonal
Original	0.873734246	0.945931872	0.827460129
Ciphered	−0.011323434	−0.010079104	0.007942569

### 3.5 Robustness analysis

**FIGURE 5:**
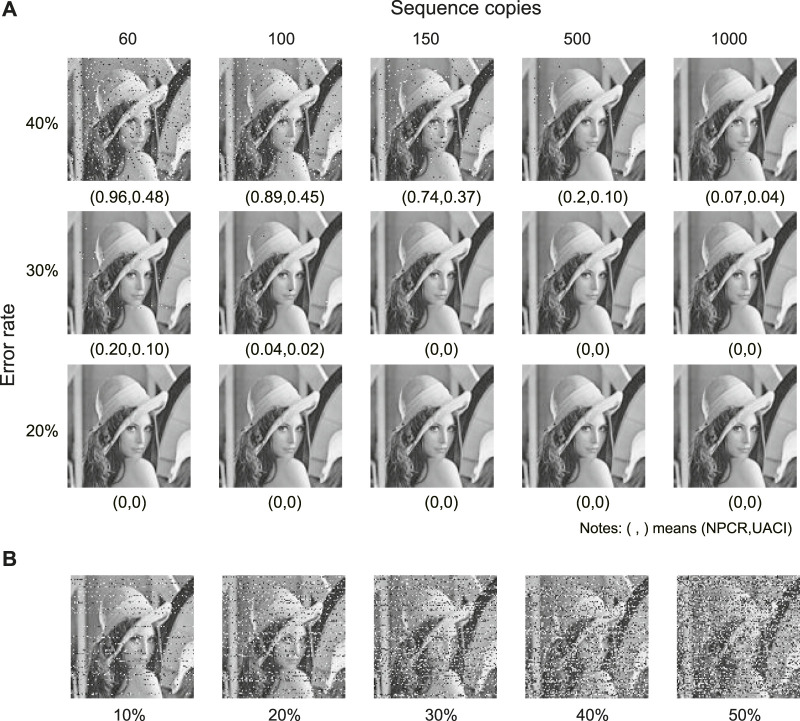
Robustness to base errors and sequence loss. **(A)** Decrypted images at different error rates and sequence copies. **(B)** Decrypted images at different sequence loss rates.

The proposed method is robust to the two most commonly seen errors in DNA storage: base errors and sequence loss. Sequence loss refers to the loss of some DNA molecules during DNA storage processes (e.g., DNA decay, PCR, and sequencing) due to the complexity of the biochemical reactions. Figure 5A shows the decrypted images at an error rate of 20 ∼40% and sequence copies 50∼1,000. The original images could be completely deciphered, given sufficient sequence copies. Figure 5B shows the decrypted images which could retain the portrait even at a loss rate of 50%. It should be added that the proposed method can easily be combined with an erasure code, such as a fountain code (Mackay, 2005), to further improve its resistance to sequence loss attacks. The combined method is quite simple. All that is required is to encode *P*_3_ with a fountain code prior to dynamic modulation encryption. To the best of our knowledge, such robustness can only be achieved by modulation-based DNA storage architecture (Yazdi et al., 2017; Antkowiak et al., 2020; Press et al., 2020; Srinivasavaradhan et al., 2021; Song et al., 2022; Zan et al., 2022).

### 3.6 Classical attack analysis

The proposed method is resistant to classical attacks, such as known plaintext attacks, chosen plaintext attacks, and chosen ciphertext attacks. As mentioned earlier, the secret keys depend not only on the given initial values, such as modulation keys and system parameters, but also on the plain image. For every plain image, the keys are changed both in the encryption process and decryption process. As such, attackers cannot extract any useful information, either by encrypting a pre-designed special image or by decrypting a certain ciphertext. This concludes that chosen plaintext, chosen ciphertext, and known plaintext attacks do not work against the proposed method.

### 3.7 Comparisons with other methods

Table 2 shows the detailed comparisons of existing studies. When compared with other methods, our method has the following advantages in terms of encryption using DNA molecules: first, the modulation key and chaotic systems feature our encryption scheme with dynamic encoding and encryption, which can withstand any kind of brute force attack. More importantly, modulation encoding provides a natural way to comply with biochemical constraints for long-term storage. Second, encrypting data by noise storage channels avoids the complexity and uncertainty in biochemical reactions, such as DNA strand displacement, DNA hiding, and DNA self-assembly. Finally, it is the only method with both high logical information density and strong robustness, which can tolerate extreme environments with high base noise and sequence loss. We believe that all these features endow our method with the potential to achieve reliable, secure, robust, and scalable encryption for DNA storage.

**TABLE 2:** Comparisons of encryption methods for DNA storage.

Literatures	Dynamic encoding	Dynamic encryption	Robustness	Biological encryption	Large-scale encryption	Logical density (bits/nt)	Key space
Yang et al. (2014)	*√*	*	*	*√*	×	0.006	$\left(\begin{array}{c} 4^{25}\times 1000\\ 2000 \end{array}\right)$
Zakeri et al. (2016)	×	*	*	*√*	×	0.239	9.1 × 10^61^
Zhang et al. (2019)	×	*	*	*√*	×	0.001	2^702^
Peng et al. (2021)	*√*	*√*	×	*√*	×	1.65	2^400^
Zhu et al. (2022)	*√*	*√*	×	*√*	×	2.0	2^1,536^
This work	*√*	*√*	*√*	*√*	*√*	1.0	2^462^

×, indication of minimum level of support; *√*, indication of acceptable level of support; *, partial fulfillment.

## 4 Conclusion

We propose an image encryption method for DNA storage which includes two parts: conventional encryption and DNA storage channel encryption. The proposed method highlights the importance of unpredicted modulation signals in a highly error-prone DNA storage channel. Simulation results show that our method is feasible and effective for encrypting and decrypting images when the error rate of the DNA storage channel is higher than 20%. There are two ways to generate such high noise: one is to adopt high-error DNA operating technologies, such as light-directed maskless array DNA synthesis, biased PCR, and nanopore sequencing; the other is to construct multiple substitution-rich copies prior to DNA synthesis with an error rate of 20% for each coding sequence. Further analysis of the security shows that it is sensitive to both keys and plaintexts, has a large enough key space, and can resist various attacks (i.e., statistical, only ciphertext, noise and data loss, etc.). When compared with other state-of-the-art encryption methods, our approach has high logical information density, compliance with biochemical constraints, and strong robustness to base errors and sequence loss; it is thus more suitable for large-scale DNA encryption storage. Although designed for image encryption, our method can also be applied to other areas of encryption. Relying on the powerful error correction capability of the modulation-based DNA storage architecture, we believe our approach will further accelerate the arrival of large-scale DNA encrypted storage.

## Data Availability

The original contributions presented in the study are included in the article/supplementary material; further inquiries can be directed to the corresponding author.
